# An Efficient Lightweight Hybrid Model with Attention Mechanism for Enhancer Sequence Recognition

**DOI:** 10.3390/biom13010070

**Published:** 2022-12-29

**Authors:** Suliman Aladhadh, Saleh A. Almatroodi, Shabana Habib, Abdulatif Alabdulatif, Saeed Ullah Khattak, Muhammad Islam

**Affiliations:** 1 Department of Information Technology, College of Computer, Qassim University, Buraydah 51452, Saudi Arabia; 2 Department of Medical Laboratories, College of Applied Medical Sciences, Qassim University, Buraydah 51452, Saudi Arabia; 3 Department of Computer Science, College of Computer, Qassim University, Buraydah 51452, Saudi Arabia; 4 Centre of Biotechnology and Microbiology, University of Peshawar, Peshawar 25120, Pakistan; 5 Department of Electrical Engineering, College of Engineering and Information Technology, Onaizah Colleges, Onaizah 56447, Saudi Arabia

**Keywords:** deep learning, enhancer sequence, convolution neural network, sequential learning models, temporal attention mechanism

## Abstract

Enhancers are sequences with short motifs that exhibit high positional variability and free scattering properties. Identification of these noncoding DNA fragments and their strength are extremely important because they play a key role in controlling gene regulation on a cellular basis. The identification of enhancers is more complex than that of other factors in the genome because they are freely scattered, and their location varies widely. In recent years, bioinformatics tools have enabled significant improvement in identifying this biological difficulty. Cell line-specific screening is not possible using these existing computational methods based solely on DNA sequences. DNA segment chromatin accessibility may provide useful information about its potential function in regulation, thereby identifying regulatory elements based on its chromatin accessibility. In chromatin, the entanglement structure allows positions far apart in the sequence to encounter each other, regardless of their proximity to the gene to be acted upon. Thus, identifying enhancers and assessing their strength is difficult and time-consuming. The goal of our work was to overcome these limitations by presenting a convolutional neural network (CNN) with attention-gated recurrent units (AttGRU) based on Deep Learning. It used a CNN and one-hot coding to build models, primarily to identify enhancers and secondarily to classify their strength. To test the performance of the proposed model, parallels were drawn between enhancer-CNNAttGRU and existing state-of-the-art methods to enable comparisons. The proposed model performed the best for predicting stage one and stage two enhancer sequences, as well as their strengths, in a cross-species analysis, achieving best accuracy values of 87.39% and 84.46%, respectively. Overall, the results showed that the proposed model provided comparable results to state-of-the-art models, highlighting its usefulness.

## 1. Introduction

Transcriptomics describes enhancers [1] as DNA segments that target the control of gene expression for the production of proteins (activators) and RNA. These proteins are comprised of transcription factors, which are proteins that are involved in the transcription process in which DNA is transformed into RNA and vice versa [2]. It is possible for these to be located as far as 1 MBp away from the gene or even at different locations on the chromosome [3]. Identifying enhancers can be difficult, since they are present in perceptible genomic sections due to their vigorous nature. Over the past few years, a number of useful methods have been developed that have helped to overcome challenges associated with identifying enhancers at the genomic level. As a result of recent research, the presence of enhancers in vertebrates and mammals has been confirmed [4]. For example, genes upregulated by inflammatory bowel disease (IBD) contain highly enriched concentrations of IBD-associated SNPs, and the transcription factors bound to the promoters and enhancers of these genes are very similar to those binding to the genes of IBD [5].

There is still a significant amount of work to be done in order to identify enhancers and correlate them with human biology and disease on a global scale. Enhancers play a significant role in determining the function of many genes throughout the human genome. Furthermore, both prokaryotes and eukaryotes feature enhancers as part of their DNA. There is a certain sequence in DNA called a promoter; it is a slightly different piece of DNA where gene transcription begins [3]. The promoter is typically found at the beginning of a gene, but an enhancer is usually found at the end of a gene, or even on a chromosome that has no genes on it. It is extremely challenging to identify new enhancers when there is such a disparity in location between these various enhancers. In certain contemporary studies of alterations, it was demonstrated that enhancers are a large family of functional elements. These may be divided into several subgroups, whose targets undergo different types of biological activities, and regulatory effects based on their target mutations [6]. There is further evidence that genetic variation in enhancers is linked to an increased risk of diseases in humans, such as inflammatory bowel disease and a variety of cancers.

Recently, significant computational work was conducted in order to identify regulator enhancers using computational algorithms. This was done as part of efforts to save time and money; experimentation is time-consuming, expensive and not always effective. The outgrowth of biological data has become a major concern for computational researchers, as they now have high-profile computing assets, as well as sophisticated strategies with which to deal with it. Several computational prediction models to rapidly recognize enhancers in genes were developed in recent years as a result of the improvement of machine learning. These include Enhancer-LSTMAtt [7], CSI-ANN [8], EnhancerFinder [9], Chrome, GKM-SVM [10], DEEP [11], GenSVM [12], RFECS [13], EnhancerDBN [14], and BiRen [15]. Despite this, these methods merely act as a classification tool for enhancers that have been identified. Meanwhile, there are many different types of enhancers, including strong and weak enhancers, pent-up enhancers, and inactive enhancers. Enhancers are broad-ranging and comprise numerous subgroups of enhancers. There is a primary predictive model that relies solely on sequence data as a starting point for distinguishing enhancers and their quality as part of a prediction tool that can assist predictors in identifying enhancers and their quality. This model is known as iEnhancer-2L [3]. In order to identify enhancers and their strengths, and categorize them accordingly based on their strength, several accurate predictors have been proposed. These include iEnhancer-EL [16] and iEnhancer-2L [3], which can identify enhancers and their strengths, as well as classify them accordingly as strong or weak enhancers. According to Liu et al., iEnhancer-2L [3] proposed a methodology which employed a support vector machine (SVM) learning algorithm to incorporate operational changes. The PseKenny-Nucleotide Composition (PseKNC) is a secondary nucleotide composition scheme that was used in addition to pseudo-k-tuple nucleotide compositions as the sequence-encoding scheme in iEnhancer-2L. In 2018, a new improved version of iEnhancer-2L called iEnhancer-EL [16] was presented as a replacement for iEnhancer-2L [3]. The first stage of this model required a set of six classifiers, while the second stage required an ensemble of ten classifiers, which made it a complex model to implement. Several elementary classifiers [16] composed of three different feature categories—the PseKNC, the k-mers, and the subsequence profiles—were used to construct the crucial classifiers. These classifiers were assembled using many SVM-based elementary classifiers [16]. As much as machine learning-based methods like those mentioned above are capable of delivering good results, it has been shown that deep learning models produce better results without requiring a manual feature extraction operation. Furthermore, machine learning techniques for genomics analysis still require input features that are hand-designed by an individual and extracted from predetermined sequences of input data in order to be able to make decisions [17,18]. It should be noted, however, that convolutional neural networks (CNNs) are able to quickly extract substantial features from a variety of stages. The iEnhancer-EL method [16] is currently considered one of the best methods for classifying enhancers and their strength, but it is likely to inspire even better models, such as ones that use new encoding methods and learning algorithms. In order to increase the accuracy of predictions of enhancer strength, we proposed to use a two-stage framework, instead of relying on a single deep learning prediction framework, in the first stage aimed at classifying enhancers. This framework was dubbed iEnhancer-Deep. Despite the fact that the aforementioned experimental methods may be useful to some extent, at the time of this writing, there did not appear to be any unified standard for the identification of enhancers in biology. Furthermore, current empirical approaches are labor-intensive, time-consuming, and impractical, rendering them ineffective for application to all cell types at various stages of the cell cycle at the same time. The model used in this paper offered the use of One-Hot encoding as well as the use of CNNs to encode sequences. There was also an effort to determine whether the input sequences from the proposed model met the quality and strength criteria to be classified as enhancers during testing. They were sent to the secondary stage to determine this. In the case that the proposed sequence did not meet all of these criteria, the sequence was referred to as a non-enhancer sequence. The analysis in this study was based on Chou’s five-step rule, which has been extensively used in recent studies [17,18,19,20]. As required, the following procedures were followed: (i) for the preparation and testing of the indicator, the large benchmark dataset must be assembled and analyzed; (ii) enhance the significance of genomic sequences by emphasizing a meaningful pattern during the extraction or determination; (iii) develop a classifier capable of identifying these sequences in an effective and accurate manner; (iv) perform various cross-validation techniques on the data; and (v) assemble a web server. To summarize, we presented a learning-based model that enabled accurate identification of enhancer sequences, their strength, and their evolutionary properties. Our article’s novel contributions can be summarized as follows:

In the current state of enhancer sequence classification, many methods rely on manual features extraction to be effective. When it comes to analyzing pre-miRNAs, there are two major approaches; one focuses on their spatial structure, while the other focuses on their sequential structure. Both are ineffective. We developed hybrid architectures that combined the encoding and representation power of convolutional neural networks (CNN) with the ability to handle large DNA sequences and the ability to accurately identify enhancers based on DNA sequence alone—making them ideal for handling DNA sequences.

There are different ways in which nucleotides can be represented within a sequence of nucleotides, such as the assignment of labels and the encoding of those labels. We were able to convert these nucleotide positions into a numerical description with the help of efficient coding methods, which we could then use to illustrate the nucleotide positions within the sequence.

The framework was validated using several benchmark enhancer sequence datasets in order to achieve state-of-the-art results for accurate classification of enhancer sequences and their strengths and prove the validity of the framework.

This manuscript was organized in the following manner: Section 2 explains the materials and methods used to identify enhancer sequences in this manuscript. In Section 3, we discussed the implementation of the proposed model, the experimental results, and the evaluation of its performance. We then discussed the conclusions of our current research in Section 4.

## 2. Materials and Methods

**Figure 1:**
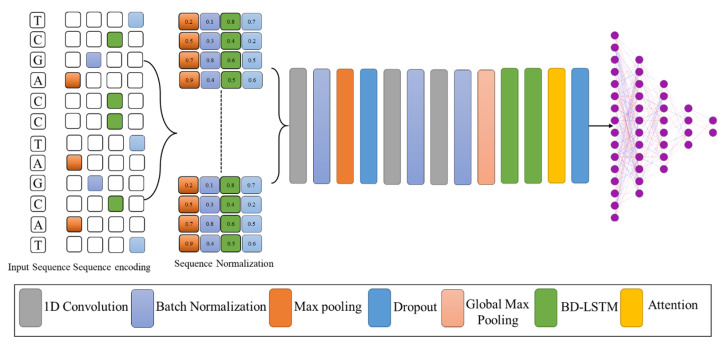
The proposed model for enhancer sequence classification and strength identification.

This section was intended to provide a brief overview of the underlying architecture of the proposed model, as illustrated in Figure 1. First, we described the basic structure of the proposed model in order to facilitate a better understanding. Next, we discussed the details of the feature extraction method in our model, which represents the encoding technique and the backbone CNN as a feature vector. In the last step, we used attentional bidirectional GRUs to map long-range dependencies on arbitrary DNA sequence lengths and form fixed-length feature representations. Table 1 presents all the abbreviation used in this work with their description.

### 2.1. Dataset

This study utilized a dataset obtained from Liu et al. in their study [3]. Furthermore, this dataset was used in the development of iEnhancer-EL [16], iEnhancer-2L [3], and EnhancerPred [21]. There were nine different cell lines in this dataset. These were used for the extraction of enhancers, which were separated from short 200 bp clips of the same length and extracted as DNA groupings out of the DNA. A total of nine different cell lines were used in this study, including H1ES, K562, GM12878, HUVEC, HSMM, NHLF, NHEK, HepG2, and HMEC. We used CD-HIT software program [22] to prevent pairwise sequences with more than 20% of features in common that were present in each sequence from crossing. In the benchmark dataset, there were 1484 enhancers, of which 742 were strong enhancers, while the remaining 742 were weak enhancers—which is an increase from the baseline dataset. Thus, based on the information provided above, the benchmark dataset can be defined as follows:

(1) $$𝒟={𝒟}^{+}\;\cup \;{𝒟}^{-}$$

(2) $${𝒟}^{+}={𝒟}_{Strong}^{+}\;\cup \;{𝒟}_{weak}^{+}$$

There are positive and negative sequences in the dataset, where 𝒟 represents the overall number of sequences in the dataset. Set theory illustrates the concept of union through the use of the symbol ∪. An enhancer subset of ${𝒟}^{+}$ contained 1484 enhancer sequences, while non-enhancer subsets of ${𝒟}^{-}$ contained 1484 non-enhancer sequences. There were 1484 enhancers in the original set, divided into two parts: the strong enhancers, which constituted 752, and the weak enhancers, which comprised 742 enhancers. There are both ${𝒟}_{Strong}^{+}$ (strong enhancers) and ${𝒟}_{weak}^{+}$ (weak enhancers) enhancers within the nine tissues emphasized above; however, there was substantial variation between tissues for ${𝒟}^{+}$ weak (weak enhancers). As a result, human embryonic stem cells were used to develop weak enhancers to account for this. The training set provided us with the opportunity to build two different models for two different problems, much like other studies have done. The first model was used to identify enhancers in stage 1, while the second model was used to classify enhancers in stage 2. The training set was divided randomly into ten folds, utilizing stratified sampling for both layers, with a randomized distribution between each fold. We used each fold individually as a validation set, then used the remaining four folds as the training set and as a basis for the construction of a CNN model using the ten folds.

### 2.2. Sequence Encoding and Proposed Model

The proposed method involved taking DNA segments with a size of 200 bp and converting them into a number sequence in which N represents the character of the unknown nucleotide. The convolution module and the Gate Recurrent Units (GRU) with attention mechanism were used to process the sequences of numbers that were entered. There were two main modules in the LSTM: a convolution module that used mainly 1D CNNs, and an attention module that used mostly feed-forward LSTMs and bi-LSTMs [23,24]. After concatenating the outputs of the two modules, the outputs were incorporated into the fully connected layer of the system. The final layer of the network followed after the fully connected layer. This latter contained two neuronal structures that represented the probabilities of belonging to enhancer layers. With a threshold of 0.5, it was predicted that a positive input would generate an output above 0.5, whereas a negative input would cause output above 0.5 to indicate the opposite. 

### 2.3. Features Extraction

Convolutional neural network architecture is one of the most common structures employed to build deep neural networks [24,25] in different domains, including fire detection [26,27]. It is mainly known for its ability to locate and capture local hidden structures by means of convolutional kernels, or filters, within the network. Convolution kernels map input feature maps into feature maps based on inputs, which in turn are further convoluted. A stride is a horizontal interval between adjacent patches that can overlap, and it is measured by the distance between adjacent patches. Each patch in the same input shares a convolutional kernel set of parameters which can be learned over time. As a result of the input padding being required in some cases, the size of the input can always be maintained without changing. In order to increase the nonlinear capability of the CNN, the activation function of the feature map can be used to increase its nonlinear capacity. The activation function can be composed of the following functions: ReLU, sigmoid, tanh, weakly ReLU, and ELU. In CNNs, the pooling function is a nonlinear down-sampling procedure whose purpose is to reduce the dimensionality of representations. This serves to speed up computations by reducing their dimension. A further advantage of the pooling technique is that it is capable of avoiding or reducing the problem of over-fitting.

### 2.4. Multi-Layer Bi Direction Gated Recurrent Units

**Figure 2:**
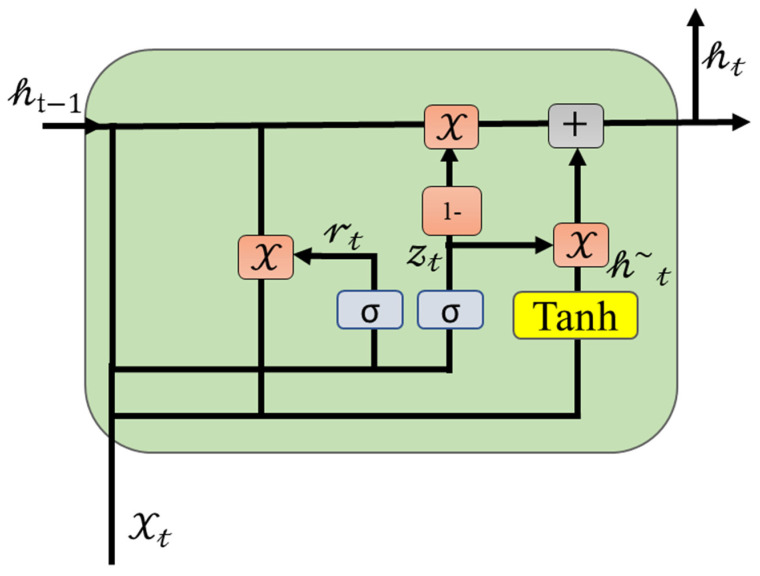
Illustration for the internal mechanism of GRU.

In the area of recurrent neural networks (RNNs), long-short term memory (LSTM) [28] is also known as a recurrent neural network (RNN) [29]. RNNs are particularly suitable for time series questions because of the way their architecture works: they share weights at every single time step in a series. Various applications of RNNs have been made in various fields, including anomaly detection [23], continuous B-cell epitope prediction [30,31], sentiment analysis [32,33], action recognition [34], and time series data analysis [35,36]. Generally, RNNs are prone to causing gradient vanishing or exploding when they are applied to long sequences of data; this is one of their major shortcomings. Consequently, RNNs were only capable of analyzing short sequences of data [37]. As a result of LSTM [28], gate mechanisms were used to control information conveying, which included selective additions and deletions of information that had already been accumulated. Although the LSTM was effective in capturing the relationship between words that were in the front and those in the back, it was unable to characterize the relationship between the words in the back and those in the front. In order to tackle this issue effectively, Bi-LSTM solutions are used [25,38]. By utilizing GRU [39], Cho proposed a new model that accounted for recurrences on different time scales, using loop blocks as an adaptive means of simplifying LSTM models without reducing their effectiveness. GRU models require the previous word vector results to be processed in order to perform the actual computation of the current word vector for a sequence $𝒮=\left[{𝒮}_{0},{𝒮}_{1},{𝒮}_{2},\;\ldots \ldots .{𝒮}_{N}\right].$ Figure 2 illustrates the GRU model that was developed. In contrast to the LSTM model, the GRU model did not contain any storage units, which was an important difference from the LSTM model. As a result of these calculations, the following result was obtained:

(3) $${𝓇}_{𝓉}=\sigma \left(W_{𝓇}\cdot \left[{𝒽}_{t-1}*X_{𝓉}\right]\right)$$

(4) $${𝓏}_{𝓉}=\sigma \left(W_{𝓏}\cdot \left[{𝒽}_{t-1}*X_{𝓉}\right]\right)$$

(5) $${𝒽}^{\sim }{}_{𝓉}=tan𝒽\left(W_{𝒽}\cdot \left[{𝓇}_{𝓉}\;*{𝒽}_{t-1}*X_{𝓉}\right]\right)$$

(6) $${𝒽}_{𝓉}=\left(1-{𝓏}_{𝓉}\right)*{𝒽}_{t-1}+{𝓏}_{𝓉}*{𝒽}^{\sim }{}_{𝓉}$$

In the equations above, ∗ is a symbol that represents the multiplication of the elements corresponding to the matrices. During the reset gate ${𝓇}_{𝓉}$, all the previous activations of the units of the same layer are taken and updated to determine the number of units being updated. Depending on its activation, the number of units that are updated from the update gate ${𝓏}_{𝓉}$ is determined. Finally, the activation unit for a GRU is generated by combining the past activation unit with the current candidate unit.

### 2.5. Attention Mechanism with GRU

The field of deep learning has increasingly focused on studying attention mechanisms. The mechanisms of attention are essentially a way of allocating weights. The scheme of distributing weights is very similar to what one does when one watches an object and places a different focus on different parts of the object. It is well known that there are several attention schemes, such as feed-forward attention [40] and self-attention [41]. In order to address the medium-term dependency related to the GRU, a feed-forward attention was used to compensate for the GRU’s deficiencies in this area. We assumed that ${𝒽}_{t}$ is the hidden state in the GRU at time step. In order to generate the context vector, the feed-forward attention method was used, as follows:

(7) $$\mathbb{C}=\overset{T}{\underset{t=1}{\sum }}{𝒶}_{t\;}{𝒽}_{t}$$

(8) $${𝒶}_{t\;}=exp\left(e_{t}\right)/\overset{T}{\underset{t=1}{\sum }}exp{\left(e_{t}\right)}^{\prime }$$

(9) $$e_{t}=\beta \left({𝒽}_{t}\right)$$

In the above equations, ${𝒶}_{t}$ presents the attention weight of the model hidden state, while ${𝒽}_{t}$ and β are the learning parameters of the proposed model.

## 3. Results and Discussion

We experimentally evaluated the proposed model using various enhancer sequence datasets in order to test its performance. The evaluation metrics we used are in general use in state-of-the-art schemes as ways to assess their effectiveness. In our experiments, the results clearly proved the success of our proposed method, providing a greater degree of precision in identifying enhancer sequences compared to existing methods.

### 3.1. Training Detail, Cross Validation and Evaluation Metrices

To implement the Enhancer-AttGRU, we used Python and TensorFlow (version 2.0) as deep learning tools. In order to assess the validity of our method, we conducted tenfold cross-validation and independent testing on a notebook computer with 32G RAM and six CPUs, each of which featured a speed of 2.60 GHz. In the training process, each epoch took about 25 s, while prediction of each sample required just 2 s using the trained Enhancer-AttGRU. Table 2 shows the number of parameters, the shape of the output, and the number of layers in the enhancement LSTM algorithm for each layer. D_S_, N_S_, and F_s_ represent total numbers of dataset samples, number of steps, and features space, respectively.

We used a tenfold cross-validation approach and independent testing in order to test the predictive performance of our presented method. In the n-fold cross-validation method, the training dataset was divided into n parts, some of which had the same size, others which had only an approximate equal size. The parts of the dataset were used to train and test the model were n−1 parts. The process was repeated n times in order to achieve the desired result. For the independent test, the training datasets were used in training the model, while the independent datasets were used in testing it. Since this was a binary classification problem, we evaluated the performance of the model using common metrics such as sensitivity (Sℕ), specificity (Sℙ), accuracy (Aℂℂ), and Matthews’ correlation coefficient (Mℂℂ), all of which were defined as follows:

(10) Sℕ=TℙTℙ+Fℕ

(11) Sℙ=TℕFℙ+Tℕ

(12) Aℂℂ=Tℙ+TℕTℙ+Fℕ+Fℙ+Tℕ

(13) Mℂℂ=Tℙ×Tℕ−Fℙ×FℕTℙ+FℕTℙ+FℙTℕ+FℕTℕ+Fℙ

In the above equations, Tℙ represents the number of true positive samples, Fℕ represents the number of false negative samples, Fℙ represents the number of false positive samples, and Tℕ represents the number of true negative samples. It is generally accepted that Sℕ, Sℙ, and Aℂℂ belong to the 0–1 range. In most cases, the Sℕ, Sℙ, and Aℂℂ fall between 0 and 1 and the Mℂℂ is between −1 and 1. In general, higher values of Sℕ, Sℙ, Aℂℂ, and Mℂℂ indicate greater efficiency. 

### 3.2. Results

**Figure 3:**
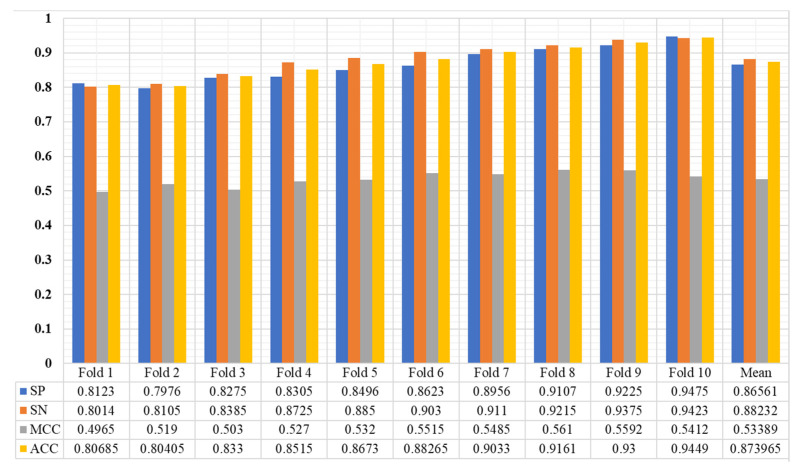
The performance of the proposed model using benchmark enhancers sequence datasets.

**Figure 4:**
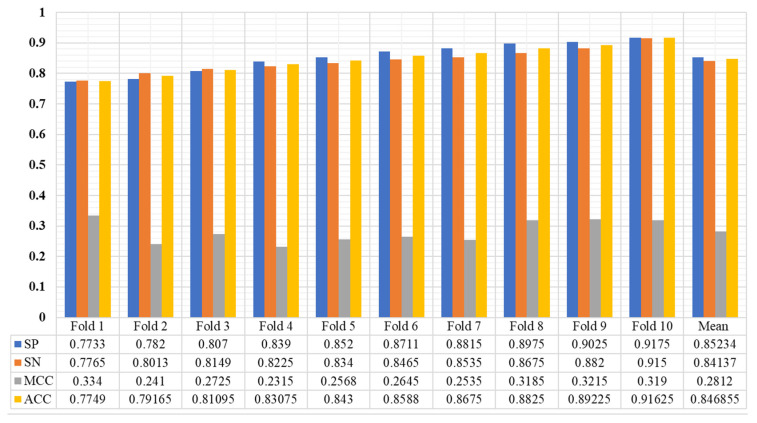
The performance of the proposed model using benchmark enhancers sequence dataset (Stage 2).

The Enhancer-AttGRU was tested to determine whether it could distinguish between enhancers and non-enhancers, as well as between strong enhancers and weak enhancers, based on its ability to identify both enhancers and non-enhancers. During the first stage of distinguishing between enhancers and non-enhancers, all of the enhancers, including weak enhancers, were considered positive samples. In the first stage, all of the enhancers were positive samples. In the second stage of the process, the strong enhancers were classified as positive, and the weak enhancers were classified as negative, a process of discriminating strong from weak enhancers. We performed a tenfold cross-validation on the dataset 𝒟 in order to evaluate the performance. In Figure 3, we presented the experimental results of the proposed model. During the first stage of the experiment, we were able to achieve an average accuracy of 0.8739%, and during the second stage, 0.8468%. As a result, the proposed model achieved Sℕ 0.8823, Sℙ 0.8656, and Mℂℂ 0.5339 in the first stage. While in the second stage the proposed model accomplished Sℕ 0.8413, Sℙ 0.8523, Aℂℂ 0.8468, and Mℂℂ 0.2804, as shown in Figure 4. It was evident that the first stage of the analysis produced much better predictive performance results than the second stage, which suggested that it was more difficult to determine whether strong enhancers were stronger or weaker than it was to determine whether non-enhancers were stronger.

### 3.3. Comparison of the Proposed Model with Existing Techniques

**Figure 5:**
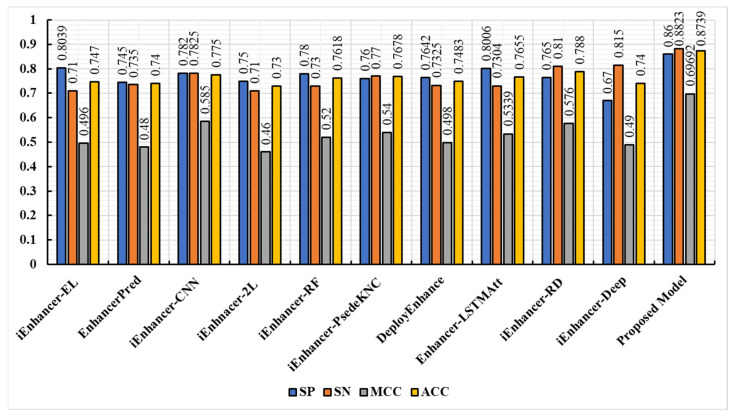
Performance comparison of the proposed model with baseline techniques (Stage 1).

**Figure 6:**
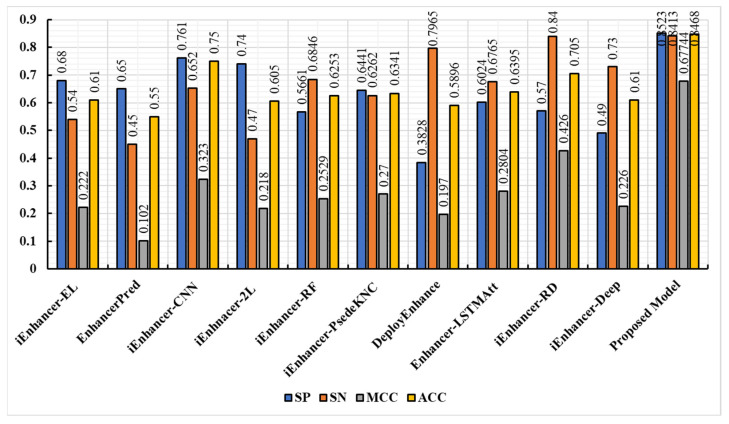
Performance comparison of the proposed model with existing techniques (stages 2).

This study evaluated the proposed model against state-of-the-art classification models, such as EnhancerPred [21], iEnhancer-RF [42], iEnhancer-PsedeKNC [43], DeployEnhance [44], iEnhancer-EL [16], iEnhancer-RD [45], Enhancer-LSTMAtt [7], iEnhancer-XG [46], iEnhancer-2L [3], iEnhancer-5Step [47], iEnhancerDSNet [48], and iEnhancer-CNN [49]. The proposed model was compared to all of these prediction methodologies in order to make a more accurate comparison. As in aforementioned studies, both of these studies used the same benchmark dataset to do their functional evaluations and analyze the data collected during the course of these studies. The comparison results showed that our model was significantly more accurate than other models. The performance of the state-of-the-art predictors in the first stage is depicted in Figure 5. A comparison of the prediction performances in the second stage is illustrated in Figure 6. In each class of metrics, the most noteworthy values are highlighted in the table below. Moreover, as illustrated in Figure 5, the performances of enhancer identification in the first stage of the algorithm were improved by 12.69%, 13.39%, 9.89%, 11.21%, 11.21%, 10.61%, 10.84%, 10.84%, 10.84%, 8.59%, and 13.39%, respectively, by resolving the enhancers in Sℕ, Aℂℂ, and Mℂℂ, respectively. Similarly, the second stage also showed significant improvements, of 23.68%, 29.68%, 09.68%, 24.18%, 22.15%, 21.27%, 25.72%, 20.73%, 14.18%, and 23.068%, respectively, as illustrated in Figure 6, for Sℕ, Sℙ, Aℂℂ, and Mℂℂ, respectively. Based on a detailed comparison between the performance of the proposed model and the performance of the existing model in our study, it was found that the proposed model achieved significant improvements in model execution. This was based on measuring the performance of the model using the performance assessment metrics. Both stages 1 and 2 demonstrated significant improvements in perceived parameters as a result of the proposed predictor, and this improvement was observed in both phases. We were able to conclude from the considerable increases in Mℂℂ values that our study provided a substantial increase in the stability of the predictor and a greater level of performance overall than that seen in state-of-the-art methodologies, which had smaller Mℂℂ values on average. As a result of this advancement, binary classification problems can now be verified to be consistent. A comparison between the Mℂℂ value and the Aℂℂ value indicated that the Mℂℂ value demonstrated a greater level of insight, in that it considered the extent of each of the four parameters (Tℕ, Tℙ, Fℙ, and Fℕ) of the confusion matrix. This demonstrated a balanced assessment during model evaluation [50]. The results of iEnhancer-Deep showed that it was capable of producing results similar to other, previously proposed strategies.

### 3.4. Experimental Result on Independent Dataset

**Figure 7:**
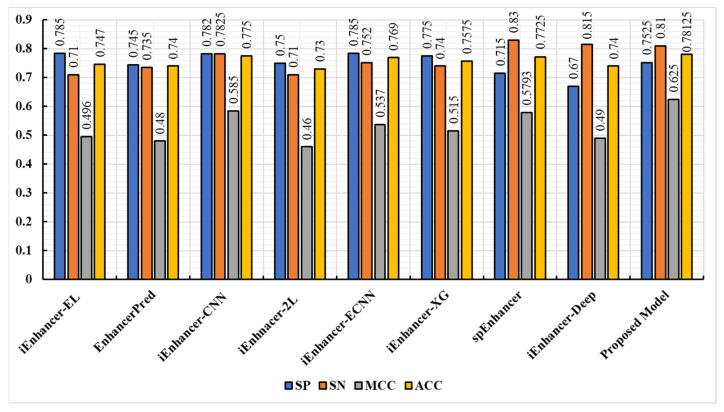
The performance of state-of-the-art predictors and the proposed model in the independent test in the first stage.

**Figure 8:**
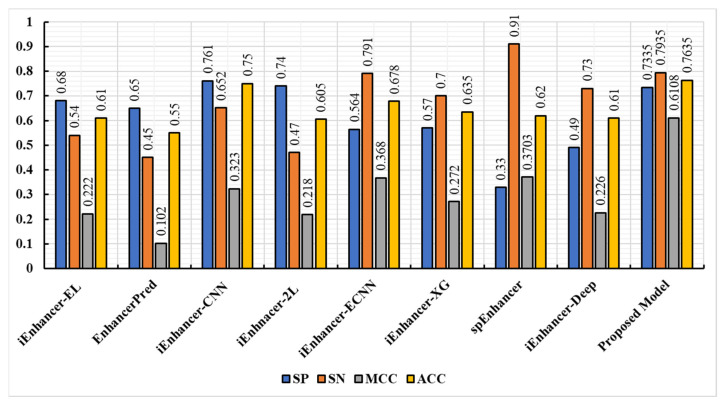
Comparison of the proposed model’s performance against state-of-the-art predictors in the second stage.

The proposed model was further tested using the independent dataset that was also presented in [16]. In this dataset, there were 100 weak enhancers, 100 strong enhancers, and 200 non-enhancers. Based on the results of the first stage comparison, Figure 7 shows the proposed model’s results against other state-of-the-art models. Figure 8 reveals the results of the second stage comparison. The results of the proposed model showed that, in terms of sensitivity, accuracy, and specificity, the proposed model performed better in both stages. In addition, the model was also capable of predicting the true enhancer sites and the strength of the enhancers, indicating its robustness. Generally, there was more confidence in the prediction time when iEnhancer-CNN was used in combination with the results of the proposed model.

### 3.5. Discussion 

We studied the effects of a number of non-enhancers on the methods in order to establish their effectiveness. Our sampling and mutation strategy was used to generate new non-enhancers. The sampling and mutation strategy was used because there were no non-enhancers available to generate before sampling. Three distinct sets of non-enhancers, including mutated non-enhancers and non-mutated non-enhancers, were formulated, along with three new training sets. As a result of the independent test that we conducted, our objective was to check the performance of the proposed method (trained from the new training sets) in order to assess its efficiency. There were up to 158,207 parameters that could be trained in the Enhancer-AttGRU. In the case of deep learning, the more parameters that can be trained, the greater the risk of overfitting. In order to reduce overfitting of the model, we used dropouts. Enhancer-AttGRU is a deep learning and end-to-end method that requires no feature design whatsoever, and is based on deep learning. Therefore, it avoids the use of artificial interference and complex methods of extracting or selecting features. In this regard, Enhancer-AttGRU is easier to implement than the feature-based methods. In cross-validation tests, the majority of feature-based methods did quite well, but they fared badly in independent tests, suggesting that they were not very generalizable. In this study, the performance of the Enhancer-AttGRU model was compared with that of nine state-of-the-art models. There are a number of deep learning-based techniques that use either CNNs or LSTMs for enhancer recognition, or combinations of these two techniques. It is important to keep in mind that these techniques are computationally intensive and have limited recognition capabilities.

## 4. Conclusions and Future Directions

There is a major role to be played by enhancers in regulating transcription for target genes. These must be identified in order to uncover their role. There is one fundamental issue which we have to deal with, and that is the difference between enhancers and non-enhancers. Initially, this classification was done using biological experiments, but, given the amount of time, money, and effort that is required to classify enhancers in this manner, it was not possible to perform this classification so early on. Therefore, we used a computational approach based on deep learning for the purpose of quickly distinguishing enhancers from others. The proposed model performed two tasks, namely: identifying enhancers and estimating the strength of these enhancers. The experimental results revealed that the proposed model outperformed state-of-the-art models. In contrast with state-of-the-art strategies, a comprehensive comparison with the proposed model suggested that the method was more than stable, it was also a highly effective and efficient method for identifying enhancers. In the future, we will explore sequence coding schemes, feature extraction methods, and data augmentation methods in order to further improve the predictive ability of the model, in terms of accuracy.

## Figures and Tables

**Table 1:** The list of notations and their descriptions used in this research work.

Notation	Description
𝒟	Dataset
${𝒟}^{+}$	Enhancer sequences
${𝒟}^{-}$	Non-enhancer sequence
${𝓇}_{𝓉}$	Reset gate
${𝓏}_{𝓉}$	Update gate
${𝒽}_{t}$	Hidden state
${𝒶}_{t\;}$	Attention weights
Aℂℂ	Accuracy
Sℕ	Sensitivity
Mℂℂ	Matthews’ Correlation Coefficient
Sℙ	Specificity
Tℙ	True Positive
Tℕ	True Negative
Fℙ	False Positive
Fℕ	False Negative

**Table 2:** Detailed information on input shape and number of parameters in proposed Enhancer-AttGRU model.

Layer	Output Shape	Param
Input layer	[(D_S_, N_S_, F_s_)]	0
conv1d (Conv1D))	(None, 298, 27)	459
max_pooling1d (MaxPooling1D)	(None, 99, 27)	0
dropout (Dropout)	(None, 99, 27)	0
conv1d_1 (Conv1D)	(None, 99, 27)	770
bidirectional (Bidirectional GRU)	(None, 256)	110,592
Attention (None, 64)	(None, 256)	0
Dropout_1 (Dropout)	(None, 256)	0
Dense (Dense)	(None, 128)	32,896
Dense_1 (Dense)	(None, 64)	8256
Dense_2 (Dense)	(None, 64)	4160
Dense_3 (Dense)	(None, 16)	1040
Dense_4 (Dense)	(None, 2)	34

## Data Availability

https://github.com/Saeedkhattak194/Enhancer-AttGRU, accessed on 4 November 2022.
